# Supplementary material for: Simultaneous single-qubit driving of semiconductor spin qubits at the fault-tolerant threshold
Source: Nat Commun. 2023 Jun 19;14:3617. doi: 10.1038/s41467-023-39334-3 (PMC10279658; doi:10.1038/s41467-023-39334-3)
Supplement: Supplementary file 1 — Supplementary Information [file 41467_2023_39334_MOESM1_ESM.pdf]

Simultaneous single-qubit driving of semiconductor spin qubits at the fault-tolerant threshold

W. I. L. Lawrie,[1] M. Rimbach-Russ,[1] F. van Riggelen,[1] N. W. Hendrickx,[1]
S. L. de Snoo,[1] A. Sammak,[2] G. Scappucci,[1] J. Helsen,[3] and M. Veldhorst[1]

[1]*QuTech and Kavli Institute of Nanoscience, Delft University of Technology, Delft, The Netherlands*
[2]*QuTech and Netherlands Organisation for Applied Scientific Research (TNO), Delft, The Netherlands*
[3]*QuSoft and CWI , Amsterdam, The Netherlands*

Supplementary Information

Contents

1. Initialization and Readout 2

2. Tuning Procedure for Single-Qubit and Simultaneous-Qubit Driving 3

3. Effects of Driving Power 5

4. Clifford Gate Set 6

5. Even and odd-parity input states for two-copy RB 7

6. Two-copy benchmarking Characterization 8

7. Decay structure of two-copy benchmarking 9
   A. Structure of the matrix decays 10
   B. Different types of two-copy RB 11
   C. Even same-system (ES) two-copy RB 11
   D. Odd same-system (OS) two-copy RB 12
   E. Even different-system (ED) two-copy RB 13

8. Two-copy RB in practice 13
   A. Extracting decay rates 13
   B. Average fidelity lower bound 14
   C. Direct comparison of two-copy benchmarking and two-qubit simultaneous benchmarking 15
      1. Simulation of the Clifford gate superoperator 15
      2. Simulation of the Clifford experiments 16
      3. Comparison between the results 16

9. Fidelity Tables 18

References 21

Supplementary Note 1: Initialization and Readout

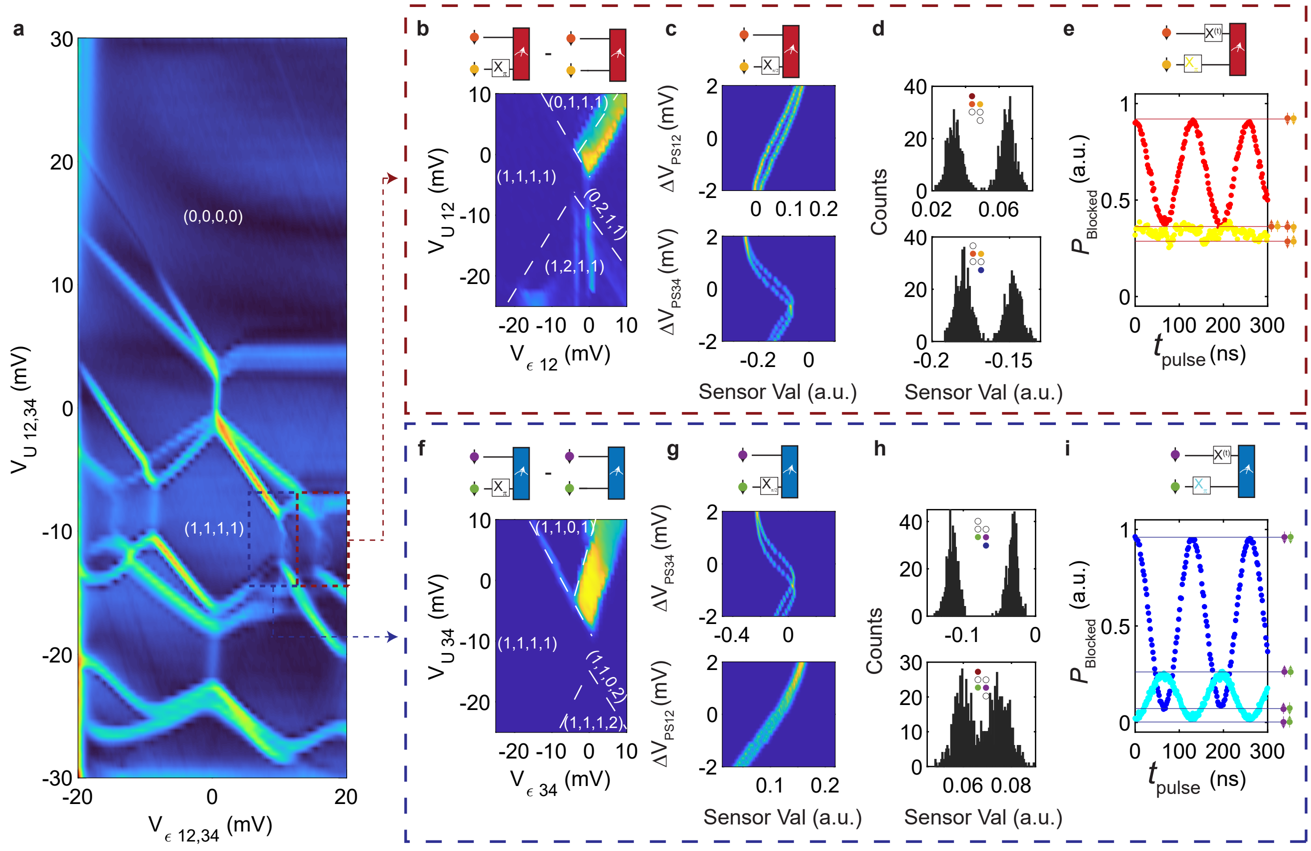

Supplementary Figure 1. (a) Charge stability diagram of the 2x2 quantum dot array. Anticrossings between the (1,1,1,1)/(0,2,1,1) and (1,1,1,1)/(1,1,0,2) charge transitions are shown in red and blue respectively. These anticrossings are used for spin-to-charge conversion and thus for readout of the qubit state. The axes $\epsilon_{12,34}$ and $U_{12,34}$ are virtual gates describing the combined detuning and energy of the two qubit pairs Q1Q2 and Q3Q4. (b) Readout calibration of the Q1Q2 system. While in the (1,1,1,1) charge state, we prepare the qubit system in the $|\downarrow\downarrow\downarrow\downarrow>$ state and apply a π-pulse to qubit Q2, bringing the system to the $|\downarrow\uparrow\downarrow\downarrow>$ state. We then sweep the detuning ($V_{\epsilon\ 12}$) and on-site energy ($V_{U\ 12}$) around the (1,1,1,1)/(0,2,1,1) anticrossing. We then repeat the experiment without applying the X_π-pulse to Q2, and plot the difference of the two measurements, and pick the point of greatest visibility as the readout position. (c-d) Single shot readout calibration. (c) Sensor signal as a function of virtual plunger gate potential (V_{PS12}) for upper (red) and lower (blue) sensors, for the Q1Q2 system. Qubit Q2 is brought into a superposition state by applying a $X_{\pi/2}$-pulse, and the system is pulsed to the readout point from (b). We repeat 1000 single shot measurements, for each value of V_{PS12} and plot the resulting histogram of sensor response. A double-gaussian is fitted to the line cuts to extract sensor response of the blocked and non-blocked signals to find the gate voltage V_{PS12} for the maximal visibility. (d) The same experiment performed at the given V_{PS12}. A double gaussian is fit to the histogram, to find the single-shot threshold, defined to be the point of equal separation. (e) Visibility of the two qubit subspace of the Q1Q2 system. Rabi oscillations are performed on Q1 with Q2 prepared either in spin-down (red) or spin-up (yellow). There is a small blocked fraction difference between the $|\downarrow\uparrow>$ and $|\uparrow\downarrow>$ states, and the $|\uparrow\uparrow>$ state is less blocked than the $|\downarrow\uparrow>$. (f-i) The same procedure as (b-e), but for the Q3Q4 system, (1,1,1,1)/(1,1,0,2) anticrossing, with X_π- and $X_{\pi/2}$-pulses on qubit Q4.

Supplementary Note 2: Tuning Procedure for Single-Qubit and Simultaneous-Qubit Driving

Tuning the resonance frequency f and Rabi period $2t_\pi$ for each single qubit for single qubit randomized benchmakring is detailed in Supplementary Figure 2a. Here, Each individual qubit is tuned by first finding f by applying an X_π pulse over a range of frequencies and fitting the optimum value. Then, X(t)-pulses at the extracted resonance frequency f are applied for varying pulse lengths t, resulting in Rabi oscillations. These are then fit to a decaying sinusoid of the form $P_{\text{blocked}} = A\exp\{-t/T_{\text{Rabi}}\}\cos(2\pi t/t_\pi + \phi) + c$ where A and c represent visibility parameters, and T_{Rabi} is the Rabi coherence time. We note that $\exp\{-t/T_{\text{Rabi}}\} \approx 1$ in all cases due to T_{Rabi} being very high for all qubits, and can be left out of the fitting routine.

Supplementary Figure 2b shows the tuning procedure for simultaneously driven qubit resonance frequencies and Rabi periods. We tune the simultaneous resonance frequencies of each qubit pair separately. This is done by preparing the two qubits in the $|{\downarrow\downarrow}\rangle$ state, applying an $\mathrm{X}_{2\pi}$ pulse each qubit simultaneously, and sweeping the IQ-modulation frequency of each qubit. The optimal simultaneous qubit frequencies are given by those which maximize return probability to the $|{\downarrow\downarrow}\rangle$ state, as indicated by a bright yellow feature in Supplementary Figure 2b. In order to tune the simultaneous qubit Rabi periods, the microwave power for each qubit is set to give both qubits the same single-qubit Rabi period (see Supplementary Figures 2 and 3). The qubits are then prepared in the $|{\downarrow\downarrow}\rangle$ state, and an $\mathrm{X}_{2\pi}$ pulse is applied to both qubits simultaneously, while varying the IQ modulation amplitude Amp. The optimal amplitudes are extracted where the return probability is maximised.

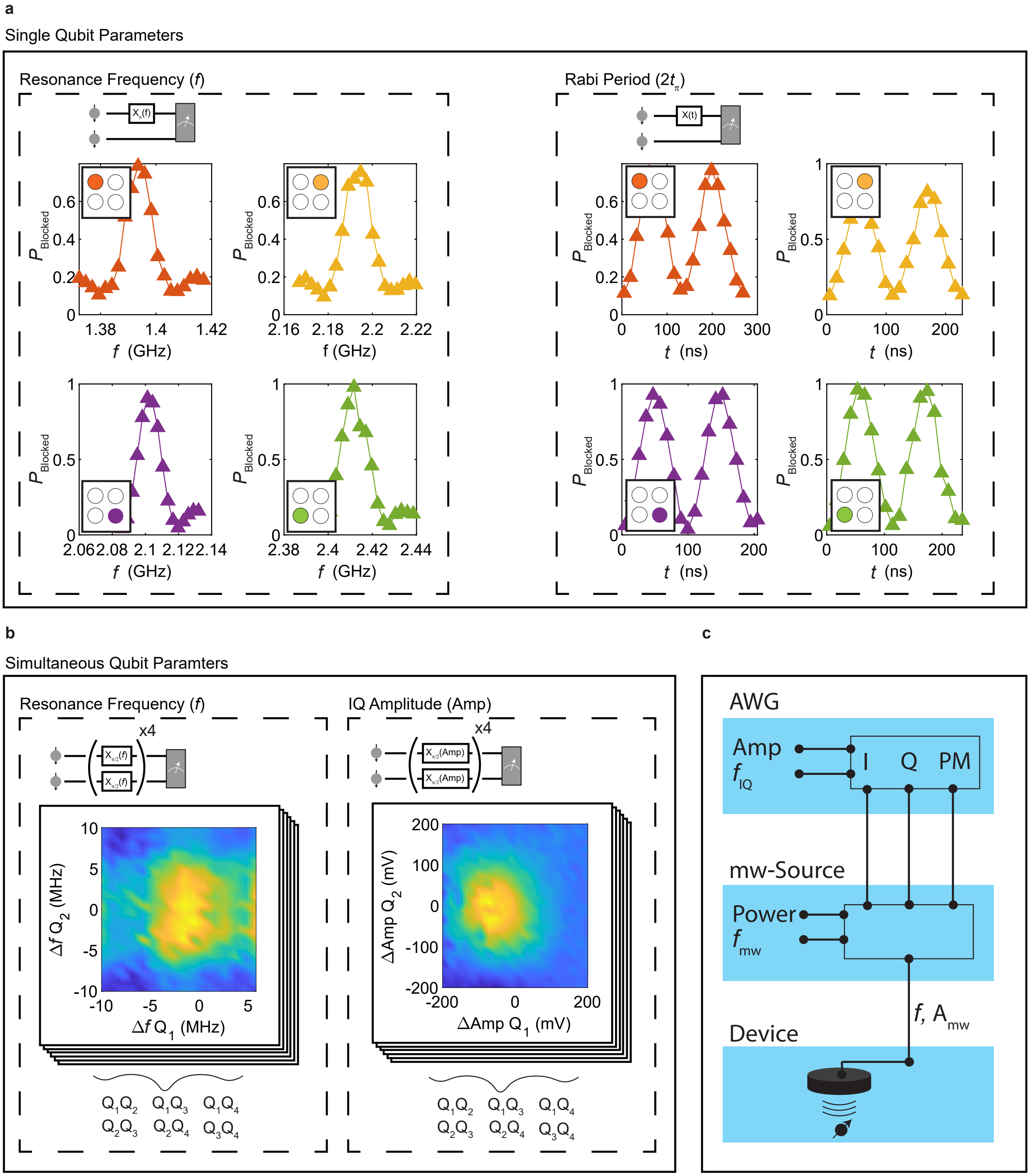

Supplementary Figure 2. Tuning protocols for single- and simultaneous-driving. (a) Single-qubit resonance frequency and Rabi period tuning protocols. (b) Simultaneous qubit tuning protocols. (c) IQ mixing schematic detailing tuning parameters.

Supplementary Note 3: Effects of Driving Power

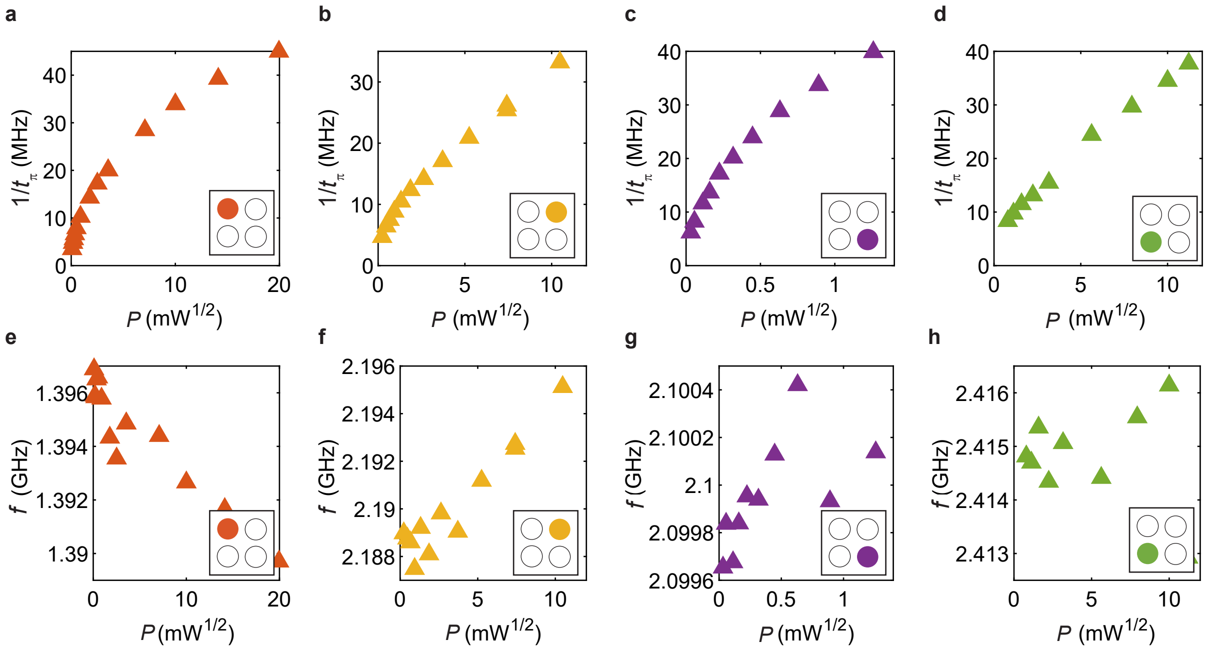

Supplementary Figure 3. Power dependence of resonance frequency shifts and $1/t_\pi$ for single qubit tuning. Data in this figure are from Main Figure 2. The calibration procedure is shown in Supplementary Figure 2a.(a-d) Extracted frequencies $1/t_\pi$ at which an X_π rotation of qubits Q1-4 is performed, respectively. (e-h) Resonance frequency dependence on driving power. The deviation in resonance frequency is likely due to an average change in position of the quantum dot due to the electric field modulation during driving, which in combination with a strong Rashba spin orbit interaction, can alter the frequency eigenstates of the qubits.

Supplementary Note 4: Clifford Gate Set

Supplementary Table I. single-qubit Clifford sequences and their composition via the minimal generator set. We benchmark by selecting a random sequence of Cliffords from the table below excluding C_1, and calculate the recovery Clifford that projects the system back into it's original state. We only use a gate set containing $\pi/2$ rotations around the Bloch Sphere, so the gates $X_{\pi/2}$ and $Y_{\pi/2}$ are explicitly referring to a rotation of $\pi/2$ around the x-axis and y-axis of the Bloch sphere of a single-qubit respectively. A $Y_{\pi/2}$ rotation is just an $X_{\pi/2}$ rotation with a $\pi/2$ software phase correction. There are on average 3.217 generators per Clifford composition. The extracted fidelity then corresponds exactly to the $\pi/2$ rotation fidelity.

**Clifford**	**Composition**
C_1	I
C_2	$Y_{\pi/2}$
C_3	$X_{\pi/2}$
C_4	$Y_{\pi/2}X_{\pi/2}$
C_5	$X_{\pi/2}Y_{\pi/2}$
C_6	$Y_{\pi/2}Y_{\pi/2}$
C_7	$X_{\pi/2}X_{\pi/2}$
C_8	$Y_{\pi/2}X_{\pi/2}X_{\pi/2}$
C_9	$Y_{\pi/2}Y_{\pi/2}X_{\pi/2}$
C_{10}	$Y_{\pi/2}X_{\pi/2}Y_{\pi/2}$
C_{11}	$Y_{\pi/2}Y_{\pi/2}Y_{\pi/2}$
C_{12}	$X_{\pi/2}X_{\pi/2}X_{\pi/2}$
C_{13}	$X_{\pi/2}X_{\pi/2}Y_{\pi/2}$
C_{14}	$X_{\pi/2}Y_{\pi/2}Y_{\pi/2}$
C_{15}	$Y_{\pi/2}X_{\pi/2}Y_{\pi/2}Y_{\pi/2}$
C_{16}	$Y_{\pi/2}Y_{\pi/2}X_{\pi/2}Y_{\pi/2}$
C_{17}	$X_{\pi/2}X_{\pi/2}X_{\pi/2}Y_{\pi/2}$
C_{18}	$Y_{\pi/2}X_{\pi/2}X_{\pi/2}X_{\pi/2}$
C_{19}	$Y_{\pi/2}Y_{\pi/2}X_{\pi/2}X_{\pi/2}$
C_{20}	$X_{\pi/2}Y_{\pi/2}Y_{\pi/2}Y_{\pi/2}$
C_{21}	$Y_{\pi/2}Y_{\pi/2}Y_{\pi/2}X_{\pi/2}$
C_{22}	$Y_{\pi/2}Y_{\pi/2}Y_{\pi/2}X_{\pi/2}Y_{\pi/2}$
C_{23}	$Y_{\pi/2}X_{\pi/2}X_{\pi/2}X_{\pi/2}Y_{\pi/2}$
C_{24}	$Y_{\pi/2}X_{\pi/2}Y_{\pi/2}Y_{\pi/2}Y_{\pi/2}$

Supplementary Note 5: Even and odd-parity input states for two-copy RB

a b

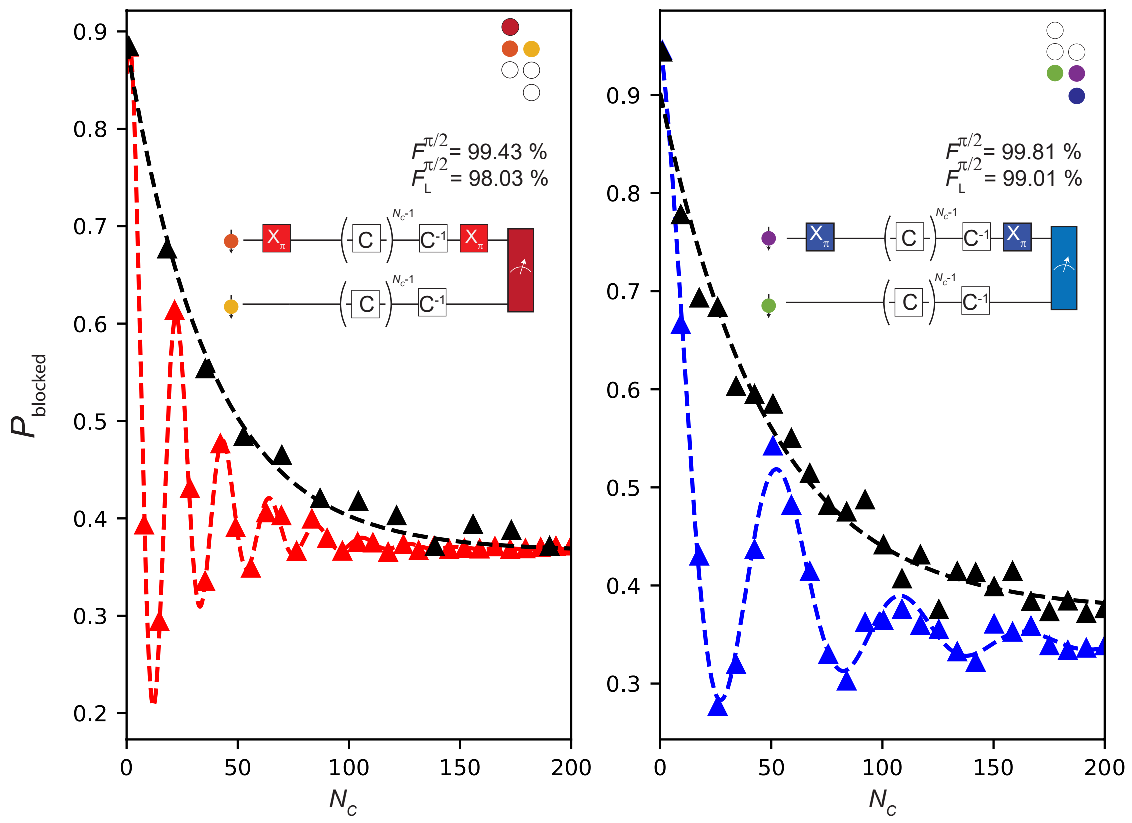

Supplementary Figure 4. ES and OS two-copy randomized benchmarking of qubits Q1 and Q2 (a) and qubits Q3 and Q4 (b) at B_{ext} = 0.65 T for the even-parity input state $|\downarrow\downarrow\rangle$ (black) and odd-parity input state $|\uparrow\downarrow\rangle$ ((a),red and (b),blue). To prepare the odd-parity space, we apply a X_π pulse to flip Q1(Q3) in the Q1Q2(Q3Q4) system before performing two-copy randomized benchmarking, then applying a second X_π pulse to the same qubit to project the final system back to the $|\downarrow\downarrow\rangle$ state. The fidelity $F^{\pi/2}$ is obtained by fitting an exponential decay to the two-copy ES RB trace (black). A rigorous lower bound on the fidelity $F_L^{\pi/2}$ can be obtained from the extracted decay rates of both even-parity (Table II) and odd-parity (Table III) experiments, by substituting them into Equation 38.

We perform ES two-copy RB on systems Q1Q2 (a) and Q3Q4 (b) for even-parity $|\downarrow\downarrow\rangle$ and odd-parity $|\uparrow\downarrow\rangle$ input states. The resulting decays give us access to every eigenvalue required to estimate the average fidelity of the system, bar one. However, as shown in section 8 B, we can place a rigorous lower bound on the average fidelity using equation (38), assuming we have experimental access to the remaining eigenvalues. Figures 4 a and B_{ext} show Two-copy randomized benchmarking experiments on combined systems Q1Q2 and Q3Q4 respectively. In these experiments, we perform two-copy RB for an even-parity input state $|\downarrow\downarrow\rangle$ (Yellow trace in (a), Cyan trace in (b)) and an odd-parity input state $|\uparrow\downarrow\rangle$ (red in (a), blue in (b)). We find that fitting a single exponent with one real decay rate to the even-parity input traces is sufficient, while for the odd-parity case, we fit three exponentials comprising one real decay and two complex conjugate decays, that give us access to decay rates λ_{adj} and $\bar{\lambda}_{adj}$ from equation (2). In the case of the real decay rates, the form of the even input states in equation (16) should access all real decay rates, while for the odd-parity case, there will be no contribution to the decay from f_{adj}. We also have no a-priori reason to know which decay is represented by which eigenvalue. However, by choosing the lower of the real decays for substitution into equation (38), we can be certain that we are lower bounding the fidelity. Tables II and III show the extracted decay rates for systems Q1Q2 and Q3Q4 respectively. By taking the lower of the real decays extracted from the even and odd-parity input state cases, we find lower bounds on the average fidelities of $F^{\pi/2}_{Q1Q2} \geq 98.03\%$ and $F^{\pi/2}_{Q3Q4} \geq 99.01\%$.

Input State	Decay	Value
$\|\downarrow\downarrow\rangle$	f_{tr}, f_{adj}, f_3	0.9722
$\|\uparrow\downarrow\rangle$	f_{tr}, f_3	0.9798
$\|\uparrow\downarrow\rangle$	λ_{adj}	0.9200+0.2779i
$\|\uparrow\downarrow\rangle$	$\bar{\lambda}_{adj}$	0.9200-0.2779i

Supplementary Table II. Measured decay rates for system Q1Q2, for even and odd-parity input states in the two-copy benchmarking experiment presented in Figure 4a.

Input State	Decay	Value
$\|\downarrow\downarrow\rangle$	f_{tr}, f_{adj}, f_3	0.9709
$\|\uparrow\downarrow\rangle$	f_{tr}, f_3	0.9770
$\|\uparrow\downarrow\rangle$	λ_{adj}	0.9715+0.1110i
$\|\uparrow\downarrow\rangle$	$\bar{\lambda}_{adj}$	0.9715-0.1110i

Supplementary Table III. Measured decay rates for system Q3Q4, for even and odd-parity input states in the two-copy benchmarking experiment presented in Figure 4b.

Supplementary Note 6: Two-copy benchmarking Characterization

Supplementary Table IV. Extracted error rates from figure 3a-b in the main text, a two-copy randomized benchmarking experiment with single, two and four-copy qubit error rates and leakage rates (when applicable) reported. Qubits measured simultaneously in the same readout system (ES) are not measured independently, so the reported number is a combined system error rate ($\epsilon_{Q1|Q2} = \epsilon_{Q2|Q1}$ and $\epsilon_{Q3|Q4} = \epsilon_{Q4|Q3}$ in the table). For this dataset, t_π = 96 ns. Leakage rates $L_{Q_i|Q_j}$ are reported when the decay plateau approaches the expected value of the fully depolarized two-qubit subspace, and are taken to be the longer of the two decay constants. The reported error rates $\epsilon_{Q_i|Q_j}$ indicate a leakage free fidelity, such that the true fidelity can be calculated as F=1-L-ϵ (see methods). Note that both two-copy ES RB and two-copy ED RB errors are reported in the table. The two-copy ES RB experiments are highlighted in purple, while the ED RB experiments are highlighted in green. The quoted errors correspond to the fit uncertainty of the traces.

	**Q1**	**Q2**	**Q3**	**Q4**
ϵ_{1Q}	0.000329 ± 0.000042	0.003155 ± 0.000310	0.000308 ± 0.000019	0.003224 ± 0.000446
L_{1Q}	-	-	-	0.000153 ± 0.000041
$\epsilon_{Q_i\|Q1}$	-	0.004018 ± 0.000130	0.000249 ± 0.000022	0.003087 ± 0.000690
$\epsilon_{Q_i\|Q2}$	0.004018 ± 0.000130	-	0.003791 ± 0.000300	0.004692 ± 0.000978
$\epsilon_{Q_i\|Q3}$	0.000301 ± 0.000048	0.003445 ± 0.000294	-	0.002778 ± 0.000298
$\epsilon_{Q_i\|Q4}$	0.002676 ± 0.000565	0.007324 ± 0.000388	0.002778 ± 0.000298	-
$L_{Q_i\|Q1}$	-	-	-	0.000175 ± 0.000065
$L_{Q_i\|Q2}$	-	-	0.000210 ± 0.000034	0.000741 ± 0.000213
$L_{Q_i\|Q3}$	-	0.000195 ± 0.000068	-	-
$L_{Q_i\|Q4}$	0.000273 ± 0.000092	-	-	-
ϵ_{4Q}	0.098565 ± 0.009031		0.020007 ± 0.000428	

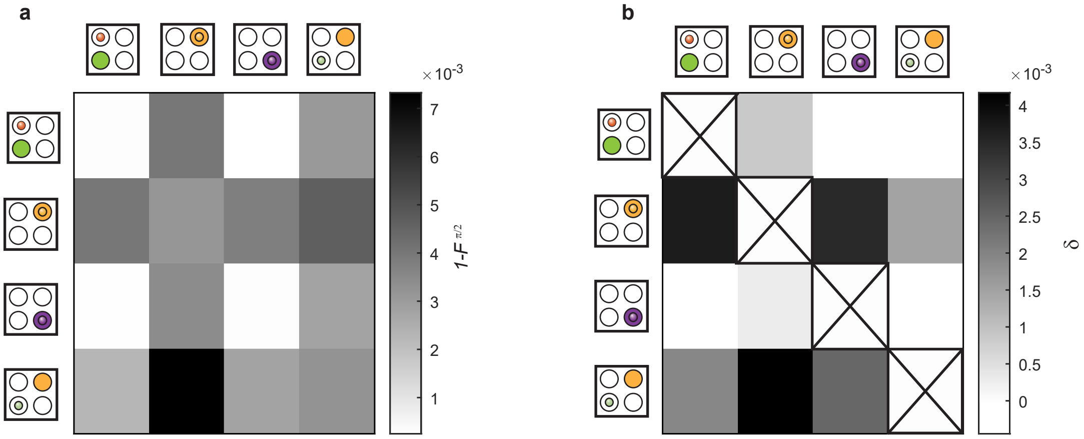

Supplementary Figure 5. Table of infidelities and relative fidelity losses of the two-simultaneously driven qubit cases reported in Table II of the supplementary information and Figure 3a-b of the main text. The plots can be interpreted by considering the qubit in question to be the column, and the qubit to be driven with to be the row. Qubits are represented diagrammatically by a small colored circle, while the plunger gate from which they are driven is depicted above them by a larger coloured circle. (a) Absolute infidelity of the simultaneously driven qubits. Values on the diagonals represent the single-qubit fidelity in the non-simultaneously driven space. (b) relative fidelity loss δ of each qubit pair. Diagonals have no significance and are set to 0. Here we observe that typically nearest neighbour qubit pairs exhibit higher values of δ, while next-nearest neighbour pairs exhibit lower values of δ. We observe some exceptions to this trend, typically when Q4 is involved in the driving. Q4 is driven via next-nearest plunger gate P_2, requiring greater power achieve a similar t_π. This is detrimental to the simultaneous driving fidelity of qubit Q2.

Supplementary Note 7: Decay structure of two-copy benchmarking

In this section we discuss the functional form (w.r.t. the sequence length) of two-copy RB data. We know from [1] that under general Markovian noise conditions the functional form of RB is fixed by the irreducible subrepresentations of the implemented representation. For one-qubit Clifford RB this representation is $R_c^1(\rho) = U_c \rho U_c^\dagger$, where ρ is an arbitrary one-qubit density matrix and U_c is the standard circuit representation of the Clifford group element c. This representation has two inequivalent irreducible subrepresentations, leading to the well-known single exponential decay seen in standard RB. In two copy-RB on the other hand the representation structure is more sophisticated. Formally we are interested in the representation structure of the representation $R_c^2(\rho) = (U_c)^{\otimes 2} \rho (U_c^\dagger)^{\otimes 2}$, where ρ is now a two-qubit density matrix. The irreducible subrepresentations of this representation were worked out in [2] (see also [3, 4]), where it was proven that R^2 decomposes into four inequivalent irreducible subrepresentations: the (one dimensional) trivial representation (appearing with multiplicity 2), the 3 dimensional adjoint representation (appearing with multiplicity 3) and two more inequivalent representation of dimensions 2 and 3 respectively (both appearing with multiplicity one). With this information we can conclude immediately from Theorem 1 in [1] that (if the noise is time-independent and Markovian) the functional form of two-copy RB data is of the form

$$p(N_C) \approx \mathrm{tr}\Big\{A_{\mathrm{tr}} M_{\mathrm{tr}}^{N_C}\Big\} + \mathrm{tr}\Big\{A_{\mathrm{adj}} M_{\mathrm{adj}}^{N_C}\Big\} + A_3 f_3^{N_C} + A_4 f_4^{N_C}, \tag{1}$$

where $A_{\mathrm{tr}}, M_{\mathrm{tr}}$ are 2×2 real matrices (associated to the trivial representation, appearing with multiplicity 2), $A_{\mathrm{adj}}, M_{\mathrm{adj}}$ are 3×3 real matrices (associated to the adjoint representation, appearing with multiplicity 3), and A_3, f_3 and A_4, f_4 are real numbers associated to the third and fourth irreducible sub-representations respectively. This decay model holds under general conditions, but to further analyse it, it is convenient to make the standard RB assumption that the noise associated to each gate in the Clifford group is constant (the so-called gate independent noise assumption). Concretely this means that we assume there exists a two qubit quantum channel Λ such that the noisy implementation of the gate $U_c^{\otimes 2}$ is of the form $\Lambda \circ R_c^2$. With this assumption we can interpret the (possibly matrix valued) decay rates seen in the two-copy RB experiment. Firstly, it follows from standard RB theory that the conventional average fidelity of the channel Λ is given as:

$$\frac{4F_{\mathrm{avg}}(\Lambda) - 1}{3} = \frac{1}{15}\big((\mathrm{tr}\{M_{\mathrm{tr}}\} - 1) + 3\,\mathrm{tr}\{M_{\mathrm{adj}}\} + 2f_3 + 3f_4\big). \tag{2}$$

Hence in principle we can learn the average fidelity by fitting the decay model in equation (1) (as in standard RB). The presence of multiple decays and matrix valued decays complicates this straightforward procedure however, and additional analysis is needed.

To do this we first need more information on the irreducible sub-representations of R_c^2. By noting that the Pauli matrices form an orthogonal basis (under the trace inner product) we can write down explicit orthonormal bases for the spaces carrying the representations discussed above (following [2]):

$$V_{\mathrm{tr},1} = \mathrm{Span}\left\{\frac{1}{2}II\right\} \tag{3}$$

$$V_{\mathrm{tr},2} = \mathrm{Span}\left\{\frac{1}{2\sqrt{3}}(XX+YY+ZZ)\right\} \tag{4}$$

$$V_{\mathrm{adj},1} = \mathrm{Span}\left\{\frac{1}{2}IX,\ \frac{1}{2}IY,\ \frac{1}{2}IZ\right\} \tag{5}$$

$$V_{\mathrm{adj},2} = \mathrm{Span}\left\{\frac{1}{2}XI,\ \frac{1}{2}YI,\ \frac{1}{2}ZI\right\} \tag{6}$$

$$V_{\mathrm{adj},3} = \mathrm{Span}\left\{\frac{1}{2\sqrt{2}}(XY-YX),\ \frac{1}{2\sqrt{2}}(ZY-YZ),\ \frac{1}{2\sqrt{2}}(XZ-ZX)\right\} \tag{7}$$

$$V_3 = \mathrm{Span}\left\{\frac{1}{2\sqrt{2}}(XX-YY),\ \frac{1}{2\sqrt{6}}(XX+YY-2ZZ)\right\} \tag{8}$$

$$V_4 = \mathrm{Span}\left\{\frac{1}{2\sqrt{2}}(XY+YX),\ \frac{1}{2\sqrt{2}}(ZY+YZ),\ \frac{1}{2\sqrt{2}}(XZ+ZX)\right\}. \tag{9}$$

In the Pauli transfer matrix representation (or Liouville representation), we can define superoperators

$$T^{i,j}_{\mathrm{rep}} = \sum_{\substack{v\in\mathrm{Basis}(V_{\mathrm{rep},i})\\ w\in\mathrm{Basis}(V_{\mathrm{rep},j})}} v\,\mathrm{Tr}\{w\,\cdot\}, \tag{10}$$

where rep can be $\mathrm{tr}, \mathrm{adj}$ and i, j run over the multiplicity of the representation labeled by rep. Note that $T^{i,i}_{\mathrm{rep}} = P^{i,i}_{\mathrm{rep}}$ is the orthogonal superoperator projector onto the space $V_{\mathrm{rep},i}$. With this definition we can, from standard RB theory, extract the matrix entries of the matrices A_{rep}:

$$[A_{\mathrm{rep}}]_{i,j} = |V_{\mathrm{rep}}|^{-1}\,\mathrm{tr}\big\{ET^{i,j}_{\mathrm{rep}}(\rho)\big\}, \tag{11}$$

where ρ, E are the implemented initial state and output measurement POVM element respectively. By the same logic we have $A_3 = (1/2)\,\mathrm{tr}\{EP_3(\rho)\}$ and $A_4 = (1/3)\,\mathrm{tr}\{EP_4(\rho)\}$ (dropping the index, as these representations only appear with multiplicity one).

A. Structure of the matrix decays

Two-copy RB differs from standard RB and simultaneous RB by the presence of matrix-valued decay rates (encoded by the matrices M_{tr} and M_{adj}) . To estimate the average fidelity we need to learn the trace of these matrices. This can be done in practice by assuming that the matrices $M_{\mathrm{tr}}, M_{\mathrm{adj}}$ are diagonalisable (note that this is a rather weak assumption). The matrix-valued signal then decomposes into a linear combination of exponentials with decay rates set by the eigenvalues. This is worth addressing in more detail. We begin by treating M_{tr}. If we assume that Λ is a trace preserving quantum channel, then certain entries of M_{tr} are fixed a priori. We concretely get that

$$M_{\mathrm{tr}} = \begin{pmatrix} 1 & 0 \\ \alpha & f_{\mathrm{tr}} \end{pmatrix}, \tag{12}$$

where α and f_{tr} are real parameters. Note that only the diagonal parameter f_{tr} contributes to the average fidelity. The case of M_{adj} is more sophisticated, and has some novel features not earlier observed in benchmarking experiments. By construction M_{adj} is a 3×3 real matrix, which means it will have at least 1 real eigenvalue f_{adj} and two more eigenvalues that are either both real, or a complex conjugate pair of complex numbers. The telltale sign of the appearance of such a complex eigenvalue pair would be the appearance of damped oscillations in the two-copy randomized benchmarking data. Note from Supplementary figure 4 that we do in fact observe such oscillations in certain two-copy RB experiments. Hence we will from now on assume that we are indeed in this scenario with two complex conjugate eigenvalues $\lambda_{\mathrm{adj}}, \bar{\lambda}_{\mathrm{adj}}$ and one real eigenvalue f_{adj}.

To gain further insight into M_{adj} it is worth making a natural extra assumption on the noise channel Λ. In particular we will assume that Λ is invariant under the interchange of two qubits. Examples of such channels include a global depolarizing channel

and cross-talk due to exchange interaction (on which more later). Through a straightforward calculation we see that if Λ is invariant under the interchange of the two qubits, then $v = (1,1,0)/\sqrt{2}$ is a right eigenvector of $M_{\rm adj}$. Since this eigenvector is real, the associated eigenvector must also be real. Complementary, the subspace spanned by $w_1 = (1,-1,0)/\sqrt{2}, w_2 = (0,0,1)$ carries the pair of complex eigenvalues.

It is worth noting that this decomposition is robust against violations of the symmetry assumption we made above, as long as the complex eigenvalues are sufficiently far from being real. By this we mean that the eigenvector v and the associated real eigenvalue will be modified continuously even under a symmetry breaking perturbation. This means the analysis above holds broadly even for quantum channels that do not exactly respect the symmetry (as long as there are two complex eigenvalues). This is important as we expect the symmetry condition not to hold exactly in practice (for instance if the two qubits have different local error rates).

To summarise, under some mild assumptions, the average fidelity takes the form:

$$\frac{4F_{\rm avg}(\Lambda)-1}{3} = \frac{1}{15}(f_{\rm tr} + 3(f_{\rm adj} + \lambda_{\rm adj} + \bar{\lambda}_{\rm adj}) + 2f_3 + 3f_4). \tag{13}$$

All these parameters can in principle be extracted from decay experiments. However, what decays are visible depends on the choice of input state and output measurement. Note that this not the case in standard RB, making two-copy RB more difficult to analyse even beyond the complexity of the representation structure. In some sense this is the price we pay for an experiment with easier-to-implement gate structure. We have implemented several types of two-copy RB, distinguished by different input states and measurements. We now discuss these in greater detail.

B. Different types of two-copy RB

With the information collected above we can further discuss the types of two-copy RB experiments discussed in the main text. We distinguish three types of two-copy RB, each with different input states ρ and measurement operators E:

$$\rho \approx |00\rangle\langle 00| = (1/4)(II + IZ + ZI + ZZ), \quad E \approx |00\rangle\langle 00| = (1/4)(II + IZ + ZI + ZZ), \quad \text{(even, same-system (ES))}$$
$$\rho \approx |00\rangle\langle 00| = (1/4)(II + IZ + ZI + ZZ), \quad E \approx |0\rangle\langle 0| \otimes I = (1/2)(II + ZI), \quad \text{(even, different-system (ED))}$$
$$\rho \approx |01\rangle\langle 01| = (1/4)(II - IZ + ZI - ZZ), \quad E \approx |01\rangle\langle 01| = (1/4)(II - IZ + ZI - ZZ). \quad \text{(odd, same-system (OS))}$$

Here even/odd refers to the parity of the input state, and same/different system refers to the type of output measurement being performed. The logical fourth experiment (odd, different-system (OD)) was not performed, so we will not analyse it here. With these expressions for E, ρ we can directly calculate what the pre-factors A in equation (1) are in the ideal case for the three executed types of two-copy RB:

$$A_{\rm tr} = \frac{1}{4}\begin{pmatrix} 1 & 1/\sqrt{3} \\ 1/\sqrt{3} & 1/3 \end{pmatrix}, \qquad A_{\rm adj} = \frac{1}{12}\begin{pmatrix} 1 & 1 & 0 \\ 1 & 1 & 0 \\ 0 & 0 & 0 \end{pmatrix}, \quad A_3 = \frac{1}{12}, \quad A_4 = 0 \tag{ES}$$

$$A_{\rm tr} = \frac{1}{4}\begin{pmatrix} 1 & 0 \\ 0 & 0 \end{pmatrix}, \qquad A_{\rm adj} = \frac{1}{12}\begin{pmatrix} 1 & 0 & 0 \\ 1 & 0 & 0 \\ 0 & 0 & 0 \end{pmatrix}, \quad A_3 = 0, \quad A_4 = 0 \tag{ED}$$

$$A_{\rm tr} = \frac{1}{4}\begin{pmatrix} 1 & -1/\sqrt{3} \\ -1/\sqrt{3} & 1/3 \end{pmatrix}, \qquad A_{\rm adj} = \frac{1}{12}\begin{pmatrix} 1 & -1 & 0 \\ -1 & 1 & 0 \\ 0 & 0 & 0 \end{pmatrix}, \quad A_3 = \frac{1}{12}, \quad A_4 = 0. \tag{OS}$$

The first thing to note here is that in all three experiments $A_4 = 0$ in the absence of SPAM errors. Hence we do not expect to be able to reliably extract f_4 from the performed experiments. We note that this is a consequence of our choices of state preparation and measurement (in particular our choice to prepare and measure in stabilizer states), and one can choose more exotic (non-stabilizer state) SPAM where f_4 is visible such as $\pi/8$-rotated states. We will now discuss each experiment in more detail.

C. Even same-system (ES) two-copy RB

As can be seen above, in the ES experiment the matrices $A_{\rm tr}$ and $A_{\rm adj}$ are rank one. Hence it is not a priori clear we can reliably observe the full matrix decays (i.e. observe all the eigenvalues of the matrices $M_{\rm tr}, M_{\rm adj}$). This is worth addressing in more detail. We begin by treating $M_{\rm tr}$. Recall that under the assumption of gate-dependent trace preserving noise this matrix has a

lower-triangular form (given by equation (12)). Note that only the diagonal parameter f_{tr} contributes to the average fidelity. Using the expression for $A_{\rm tr}$ for the ES experiment we see that

$$\mathrm{tr}\Big(A_{\rm tr}M_{\rm tr}^{N_C}\Big) = \frac{1}{4}\big(1+(1/3)f_{\rm tr}^{N_C}+\alpha(1-f_{\rm tr}^{N_C})/(\sqrt{3}(1-f_{\rm tr})\big) = af_{\rm tr}^{N_C}+B \tag{14}$$

for some real constants a, b. Hence the trivial "sector" contributes only a single exponential decay to the model in equation (1). Moreover, in the high fidelity limit the constant a will be approximately $1/12$ for the ES experiment, which means that $f_{\rm tr}$ will typically be observable.

Next consider $\mathrm{tr}\Big\{A_{\rm adj}M_{\rm adj}^{N_C}\Big\}$. The symmetry assumption made above constrains the structure of the matrix $M_{\rm adj}$. In particular we see that $a := \mathrm{tr}\{P_{\rm adj,1}\Lambda P_{\rm adj,1}\} = \mathrm{tr}\{P_{\rm adj,2}\Lambda P_{\rm adj,2}\}$ and $b := \mathrm{tr}\{P_{\rm adj,2}\Lambda P_{\rm adj,1}\} = \mathrm{tr}\{P_{\rm adj,1}\Lambda P_{\rm adj,2}\}$, while $\alpha := \mathrm{tr}\{P_{\rm adj,1}\Lambda P_{\rm adj,3}\} = -\mathrm{tr}\{P_{\rm adj,2}\Lambda P_{\rm adj,3}\}$ and $\beta := \mathrm{tr}\{P_{\rm adj,3}\Lambda P_{\rm adj,1}\} = -\mathrm{tr}\{P_{\rm adj,3}\Lambda P_{\rm adj,2}\}$. Hence the matrix $M_{\rm adj}$ has the general form:

$$M_{\rm adj} = \begin{pmatrix} a & B_{\text{ext}} & \alpha \\ b & a & -\alpha \\ \beta & -\beta & t \end{pmatrix} \tag{15}$$

with $t := \mathrm{tr}\{P_{\rm adj,3}\Lambda P_{\rm adj,3}\}$. From this construction it is easy to see that $v = (1,1,0)/\sqrt{2}$ is a (left and right) eigenvector of $M_{\rm adj}$ with real eigenvalue $f_{\rm adj} = a + b$.

Note now that $A_{\rm adj} = v^T v$ in the ES experiment, and thus that $\mathrm{tr}\Big\{A_{\rm adj}M_{\rm adj}^{N_C}\Big\} = f_{\rm adj}^m$. Hence in the absence of SPAM errors the ES experiment perfectly detects the one real decay in $M_{\rm adj}$, and does not detect the two conjugate complex eigenvalues at all. Hence, if the symmetry assumption is satisfied, the presence of oscillations in the even experiment are only due to SPAM errors. However if the symmetry assumption is only approximately satisfied then the eigenvector associated to $f_{\rm adj}$ will only be approximately $v = (1,1,0)\sqrt{2}$, which means $A_{\rm adj}$ might have overlap with vectors orthogonal to v and thus pick up an oscillatory component even in the SPAM free case. It is important to realise here that as long as the deviation from symmetry is small enough the eigenvector v will perturb continuously, which means the "SPAM-free" oscillatory component will be similar in size to the change of $f_{\rm adj}$, which will, under typical experimental conditions, be much smaller than the SPAM error (simply because $f_{\rm adj}$ is typically close to 1). Hence we will neglect this component in our analysis and consider oscillations in the ES experiment to be due to SPAM errors.

To summarize, in the ES two-copy RB experiment, we expect the data to take the functional form

$$p(N_C)_{\rm ES} \approx a_{\rm tr}f_{\rm tr}^{N_C} + b_{\rm tr} + A_3 f_3^{N_C} + a_{\rm adj}f_{\rm adj}^m, \tag{16}$$

where $a_{\rm tr}, b_{\rm tr}, A_3, a_{\rm adj}$ are real numbers encoding SPAM.

D. Odd same-system (OS) two-copy RB

The discussion of the odd same-system (OS) two-copy RB experiment is largely analogous. Considering $M_{\rm tr}$ first, we see that

$$\mathrm{tr}\Big(A_{\rm tr}M_{\rm tr}^{N_C}\Big) = \frac{1}{4}\big(1+(1/3)f_{\rm tr}^{N_C}-\alpha(1-f_{\rm tr}^{N_C})/(\sqrt{3}(1-f_{\rm tr})\big) = af_{\rm tr}^{N_C}+b, \tag{17}$$

for some real constants a, b. Again in the high fidelity limit a will be approximately $1/12$ and we thus expect good visibility for $f_{\rm tr}$. For $M_{\rm adj}$ we note that in the OS type experiment we have $A_{\rm adj} = w_1^T w_1$ with $w_1 = (1,-1,0)/\sqrt{2}$. This means the OS experiment does not observe the real eigenvalue $f_{\rm ad}$ (as the corresponding eigenvector is orthogonal). Instead we find, because $\mathrm{tr}\Big\{A_{\rm adj}M_{\rm adj}^{N_C}\Big\}$ is real-valued, that

$$\mathrm{tr}\Big\{A_{\rm adj}M_{\rm adj}^{N_C}\Big\} = a_{\rm adj}(\lambda_{\rm adj}^{N_C} + \bar{\lambda}_{\rm adj}^{N_C}), \tag{18}$$

for some real parameter $a_{\rm adj}$. Thus in this experiment we expect a quite strong oscillating component in the signal. This is confirmed in practice, see Supplementary Figure 4. To summarise, in the OS two-copy experiment we expect the data to take the functional form

$$p(N_C)_{\rm OS} \approx a_{\rm tr}f_{\rm tr}^{N_C} + b_{\rm tr} + A_3 f_3^{N_C} + a_{\rm adj}(\lambda_{\rm adj}^{N_C} + \bar{\lambda}_{\rm adj}^{N_C}), \tag{19}$$

where $a_{\rm tr}, b_{\rm tr}, A_3, a_{\rm adj}$ are real numbers encoding SPAM (similar to the pre-factor and offset in standard RB).

E. Even different-system (ED) two-copy RB

Finally we discuss the case of ED two-copy RB. This experiment has a significantly simpler decay structure (but as a consequence also collects less information about error processes). Note first that

$$\mathrm{tr}\left\{A_{\mathrm{tr}}M_{\mathrm{tr}}^{N_C}\right\} = \mathrm{tr}\left\{\frac{1}{4}\begin{pmatrix}1 & 0\\ 0 & 0\end{pmatrix}\begin{pmatrix}1 & 0\\ \alpha & f_{\mathrm{tr}}\end{pmatrix}\right\} = \frac{1}{4}, \tag{20}$$

which means f_{tr} is not visible in this experiment. Furthermore, we see that $A_{\mathrm{adj}} \propto (1,0,0)^T(1,1,0)$, and hence that

$$\mathrm{tr}\left\{A_{\mathrm{adj}}M_{\mathrm{adj}}^{N_C}\right\} = a_{\mathrm{adj}}f_{\mathrm{adj}}^{N_C}, \tag{21}$$

for some real parameter a_{adj}.

To summarise, in the ED two-copy experiment we expect the data to take the functional form

$$p(N_C)_{\mathrm{ED}} \approx b_{\mathrm{tr}} + a_{\mathrm{adj}}f_{\mathrm{adj}}^{N_C} \tag{22}$$

which means the ED experiment leads, under mild assumptions on the noise channel and SPAM, to a standard single exponential decay with offset. This single exponential only gives us incomplete information on the average fidelity of the two qubit noise channel. However, it can be interpreted in relevant special cases. Consider for instance the scenario of local noise, i.e. $\Lambda = \Lambda_1 \otimes \Lambda_2$. In this scenario the matrix M_{adj} is of the general form

$$M_{\mathrm{adj}} = \begin{pmatrix} f_1 & 0 & \alpha_1\\ 0 & f_2 & -\alpha_2\\ \beta_1 & -\beta_2 & t\end{pmatrix} \tag{23}$$

where f_1, f_2 are the single qubit depolarising parameters (and α_i, β_i, t are real parameters). From this we can directly calculate that

$$\mathrm{tr}\left\{A_{\mathrm{adj}}M_{\mathrm{adj}}^{N_C}\right\} = a_{\mathrm{adj}}f_1^{N_C} \tag{24}$$

and thus that ED two-copy RB measures, in this limit, the average gate fidelity of the first qubit (note that one can equally well define ED two copy RB such that the measurement is on the second qubit, in which case the relevant parameter is f_2). We will see numerically in the next section that this interpretation holds up quite well even when Λ is not strictly of product form.

Supplementary Note 8: Two-copy RB in practice

In the previous section we discussed the general functional form of the different types of two-copy RB, and how the extracted decay rates relate to the fidelity. In this section we will discuss in more detail how to extract these decay rates and also how to interpret them. There are three problems to deal with here. The first is the question of extracting the decay rates from the data $p(N_C)$. In the next subsection we will discuss how to deal with this problem. The second problem is that none of the three performed types of two-copy RB give access to the decay rate f_4. This means that even if we perform all three experiments on the same pair of qubits we can not evaluate the two-qubit average gate fidelity directly. We can partially deal with this question by arguing that (which we do in subsection 8 B) if one has access to all other decay rates ($f_{\mathrm{tr}}, f_3, f_{\mathrm{adj}}, \lambda_{\mathrm{adj}}, \bar{\lambda}_{\mathrm{adj}}$) one can derive a very sharp lower bound on the average fidelity proper. This allows us to give rigorous lower bounds on the average fidelity in the case where both the ES and OS two-copy experiments were performed (see Supplementary Figure 4). Additionally, one can also access f_4 directly by considering an experiment with the magic state $|T\rangle = \frac{1}{\sqrt{2}}(|0\rangle + e^{i\pi/4}|1\rangle)$ as input and corresponding magic measurements. Experimentally, this particular magic state can be very easily accessed with high fidelity as it only requires a virtual-z gate (update of the microwave drive phase) for implementation.

Finally we deal with the question of how to interpret the decay rates themselves in the scenario where no rigorous statement on the average fidelity can be given (i.e. for qubit pairs where only the ES or ED experiment have been performed).

A. Extracting decay rates

Parameter estimation of multi-exponential signals, such as equations (16), (19), is a difficult problem with a storied history. We will make use of a modern algorithm for this task called ESPRIT, which comes with rigorous performance guarantees [5] and has

been used in multiple quantum information tasks such as randomized benchmarking [1], spectral tomography [6] and low-cost phase estimation [7]. This algorithm allows us to extract decay rates, but does not allow us to identify which decay rate is associated to which irreducible representation (with the exception of the complex decay rates in the odd experiment). When computing the average fidelity (equation (2)) we will take this into account by choosing the assignment of representations to observed decays that leads to the lowest possible average fidelity, hence providing a principled lower bound. Similarly, when two decay rates are close together, ESPRIT can not distinguish between these decay rates and instead reports a single decay rate that is the average of the two (this process is called clumping [5]). We will solve this issue by assigning the smallest detected decay rate to as many decay rates as are expected. This gives a lower bound estimate on the average fidelity. It turns out that in practice the observed real decay rates are quite close together, which means the errors made here are usually small.

B. Average fidelity lower bound

In the main text we claimed that even with only partial access to the decay rates composing equation (1) we can provide a lower bound on the average fidelity (given as a weighted average of these decay rates, as per equation (2)). Here we will give a proof of this claim, which follows from the complete positivity of the channel Λ. Concretely we assume we have access (through performing both ES and OS two-copy RB), to the decay rates $f_{\mathrm{tr}}, f_3, f_{\mathrm{adj}}, \lambda_{\mathrm{adj}}, \bar{\lambda}_{\mathrm{adj}}$ (i.e. only f_4 is missing). We will argue that f_4 is sharply lower bounded by the average infidelity $r(\Lambda) = 1 - F_{\mathrm{avg}}(\Lambda)$. Key to this is the following general theorem, which might be of broader interest.

Theorem 1. *Let P, Q be* commuting *n-qubit Pauli operators and consider for a quantum channel Λ the Pauli transfer matrix element $2^n \operatorname{tr}\{P\Lambda(Q)\}$. We have*

$$\left|\delta_{P,Q} - 2^{-n}\operatorname{tr}\{P\Lambda(Q)\}\right| \leq 2\frac{2^n+1}{2^n} r(\Lambda). \tag{25}$$

where $\delta_{P,Q} = 1$ if $P = Q$ and zero otherwise.

Proof. Since P, Q commute, they are diagonal in a joint basis $\{|x\rangle\}_{x\in\{0,1\}^n}$. Since the average fidelity is invariant under unitary conjugation we can, without loss of generality, take this basis to be the computational basis. Writing $P|x\rangle = p(x)|x\rangle$ and $Q|x\rangle = q(x)|x\rangle$ for the eigenvalues of P, Q we see

$$\operatorname{tr}\{P\Lambda(Q)\} = \sum_{x\in\{0,1\}^n} p(x)q(x)\operatorname{tr}\{|x\rangle\langle x|\,\Lambda(|x\rangle\langle x|)\} + \sum_{x\in\{0,1\}^n}\sum_{\substack{x'\in\{0,1\}^n,\\ x'\neq x}} p(x)q(x')\operatorname{tr}\{|x\rangle\langle x|\,\Lambda(|x'\rangle\langle x'|)\}. \tag{26}$$

We begin by bounding the second term. By noting that $\operatorname{tr}\{|x\rangle\langle x|\,\Lambda(|x'\rangle\langle x'|)\} \in [0,1]$ we see

$$\left|\sum_{x\in\{0,1\}^n}\sum_{\substack{x'\in\{0,1\}^n,\\ x'\neq x}} p(x)q(x')\operatorname{tr}\{|x\rangle\langle x|\,\Lambda(|x'\rangle\langle x'|)\}\right| \leq \sum_{x\in\{0,1\}^n}\sum_{\substack{x'\in\{0,1\}^n,\\ x'\neq x}} \left|\operatorname{tr}\{|x'\rangle\langle x'|\,\Lambda(|x\rangle\langle x|)\}\right| \tag{27}$$

$$= \sum_{x\in\{0,1\}^n}\big(1 - \operatorname{tr}\{|x\rangle\langle x|\,\Lambda(|x\rangle\langle x|)\}\big), \tag{28}$$

where we used that $\sum_{x'}\operatorname{tr}\{|x'\rangle\langle x'|\,\Lambda(|x\rangle\langle x|)\} = 1$. We can bound this directly by using Theorem 1 from [8], which states that

$$\sum_{x\in\{0,1\}^n}\big(1 - \operatorname{tr}\{|x\rangle\langle x|\,\Lambda(|x\rangle\langle x|)\}\big) \leq (2^n+1)r(\Lambda). \tag{29}$$

For the first term we have two cases. If $P = Q$ then $p(x) = q(x)$ and thus $p(x)q(x) = 1$, which means we can lower bound the first term by $2^n - (2^n+1)r(\Lambda)$ (by the above equation). On the other hand if $P \neq Q$, we know that there are exactly 2^{n-1} values of x s.t. $p(x)q(x) = 1$ and 2^{n-1} values of x s.t. $p(x)q(x) = -1$ (since P, Q must now be trace orthogonal). Labeling these two sets X_+, X_- we see that

$$\sum_{x\in\{0,1\}^n} p(x)q(x)\operatorname{tr}\{|x\rangle\langle x|\,\Lambda(|x\rangle\langle x|)\} = \sum_{x\in X_+}\operatorname{tr}\{|x\rangle\langle x|\,\Lambda(|x\rangle\langle x|)\} - \sum_{x\in X_-}\operatorname{tr}\{|x\rangle\langle x|\,\Lambda(|x\rangle\langle x|)\} \tag{30}$$

$$= \sum_{x\in X_-}\big(1 - \operatorname{tr}\{|x\rangle\langle x|\,\Lambda(|x\rangle\langle x|)\}\big) - \sum_{x\in X_+}\big(1 - \operatorname{tr}\{|x\rangle\langle x|\,\Lambda(|x\rangle\langle x|)\}\big) \tag{31}$$

$$\leq \sum_{x\in X_-}\big(1 - \operatorname{tr}\{|x\rangle\langle x|\,\Lambda(|x\rangle\langle x|)\}\big) + \sum_{x\in X_+}\big(1 - \operatorname{tr}\{|x\rangle\langle x|\,\Lambda(|x\rangle\langle x|)\}\big) \tag{32}$$

$$\leq (2^n+1)r(\Lambda). \tag{33}$$

Putting these two cases together we obtain the theorem statement. □

With this theorem we can directly lower bound f_4 in terms of the average infidelity $r(\Lambda)$:

$$f_4 = \frac{1}{24}\Big(\operatorname{tr}\{(XY+YX)\Lambda(XY+YX)\} + \operatorname{tr}\{(XZ+ZX)\Lambda(XZ+ZX)\} + \operatorname{tr}\{(ZY+YZ)\Lambda(ZY+YZ)\}\Big) \tag{34}$$

$$\geq \frac{1}{6}\Big(6(1-\frac{5}{2}r(\Lambda)) - 6\frac{5}{2}r(\Lambda)\Big) \tag{35}$$

$$\geq 1 - 5r(\Lambda). \tag{36}$$

And thus by equation (2) we see that

$$1 - \frac{4}{3}r(\Lambda) \geq \frac{1}{15}\big(f_{\mathrm{tr}} + 2f_3 + 3(f_{\mathrm{adj}} + \lambda_{\mathrm{adj}} + \bar{\lambda}_{\mathrm{adj}}) + 3 - 15r(\Lambda)\big), \tag{37}$$

which after a rearranging of terms gives

$$r(\Lambda) \leq 3\Big(1 - \frac{1}{15}\big(f_{\mathrm{tr}} + 2f_3 + 3(f_{\mathrm{adj}} + \lambda_{\mathrm{adj}} + \bar{\lambda}_{\mathrm{adj}}) + 3\big)\Big). \tag{38}$$

C. Direct comparison of two-copy benchmarking and two-qubit simultaneous benchmarking

In this section we provide numerical evidence, beyond the formal bounds given above, that the decays numbers extracted from ES and ED two-copy RB are representative of the average fidelity. We do this by directly comparing two-copy RB to standard simultaneous RB.

One drawback of standard simultaneous benchmarking is the requirement that all Clifford gates require to be equal in time to avoid synchronization problems of simultaneous Clifford operations. In general the synchronization problem can be avoided by extending short Clifford gate sequences with an idle time such that the duration of all Clifford gate sequences is equal. Fortunately, elementary gates based on single-qubit $\pi/2$-microwave pulses with individual phase control allow to construct all 24 single-qubit Clifford gates using two "real" and a up to two "virtual" z-gates (see Ref. [9] and table V). We use this Clifford-gate compilation for a direct comparison of two-copy randomized benchmarking and standard simultaneous benchmarking. For standard two-qubit simultaneous benchmarking random single-qubit Cliffords are chosen from the set with the respective recovery Clifford. Important, each Clifford gates is chosen randomly and independently with respect to the other qubit. We note here, that the discussion regarding the simulation is independent of the Clifford compilation as derived above.

1. *Simulation of the Clifford gate superoperator*

In order to efficiently yet accurately simulate the two protocols we first compute the full time-evolution superoperator for each combination of single-qubit Clifford gates on two qubits assuming quasistatic noise. In detail we solve the time-dependent Liouville–von Neumann equation for the density matrix ρ:

$$i\hbar\frac{d}{dt}\left|\rho\right\rangle = \left[H(t)\rho - \rho H(t)\right], \tag{39}$$

where $\hbar = h/(2\pi)$ is the reduced Planck constant. Here, the system consisting of two germanium hole qubits is described by the Hamiltonian following [10, 11]

$$H(t) = \mu_B \boldsymbol{B}_1 \cdot \boldsymbol{S}_1 + \mu_B \boldsymbol{B}_2 \cdot \boldsymbol{S}_2 + \boldsymbol{S}_1 \cdot \mathcal{J}\boldsymbol{S}_2. \tag{40}$$

Here, $\mathcal{J}$ is the tensorial form of the exchange interaction between Q1 and Q2, μ_B is Bohr's magneton, h the Planck constant and $\boldsymbol{S}_j = (\sigma_x, \sigma_y, \sigma_x)^T/2$ is the vector consisting of the spin matrices acting on Qj. The effective magnetic field "felt" by Qj

$$\boldsymbol{B}_1 = (b_{Q1,f_{\mathrm{Q1}}}\cos(2\pi f_{\mathrm{Q1}}t + \phi_{\mathrm{Q1}}) + b_{\mathrm{Q1},f_{\mathrm{Q2}}}\cos(2\pi f_{\mathrm{Q2}}t + \phi_{\mathrm{Q2}}), 0, g_{\mathrm{Q1}}B_z)^T \tag{41}$$

$$\boldsymbol{B}_2 = (b_{Q2,f_{\mathrm{Q1}}}\cos(2\pi f_{\mathrm{Q1}}t + \phi_{\mathrm{Q1}}) + b_{\mathrm{Q2},f_{\mathrm{Q2}}}\cos(2\pi f_{\mathrm{Q2}}t + \phi_{\mathrm{Q2}}), 0, g_{\mathrm{Q2}}B_z)^T \tag{42}$$

consists of the external magnetic field B_z multiplied by the in-plane g-factor g_{Qj} as well as contributions due to the spin-orbit interaction. Assuming an isotropic exchange interaction, $\mathcal{J}_{jk} = J_{jk}\mathbb{1}_3$ and driving frequencies $f_{\mathrm{Qj}} = \frac{\mu_B g_{\mathrm{Qj}}}{h}B_z \gg \frac{J}{h}$ the dominating

contribution of the exchange interaction is of the form $H_J = \boldsymbol{S}_1 \cdot \mathcal{J}\boldsymbol{S}_2 \approx -\frac{J}{4}\text{ZZ}$ (see Ref. [12]). Single-qubit rotations are implemented via resonant driving $f_{\text{Qj}} = \frac{\mu_B g_{\text{Qj}}}{h} B_z$ with a Rabi frequency $\Omega_{\text{Qj}} = \frac{\mu_B}{2h} b_{Qj,f_{\text{Qj}}}$. To follow the experiment we set $\Omega_{\text{Q1}} = \Omega_{\text{Q2}} = 10.6\,\text{MHz}$. We simulate cross-talk such that each qubit is affected by both driving fields (one resonant and one is off-resonant) via the amplitudes $b_{Qj,f_{\text{Q1}}}$ and $b_{Qj,f_{\text{Q2}}}$. To simplify our simulations and maximize the cross-talk effect we set $b_{Qj,f_{\text{Qk}}} = 10.6\,\text{MHz}$ for $j,k = 1,2$.

We solve Eq. (39) by iteratively computing the unitary propagator according to

$$U(t + \Delta t) = e^{-\frac{i}{\hbar}H(t)\Delta t}U(t). \tag{43}$$

We discretize $H(t)$ into N segments of length Δt such that $H(t)$ is constant in the time-interval $[t, t + \Delta t)$. For all simulation we chose $\Delta t = 10\,\text{ps}$. Furthermore, we include quasistatic noise in the simulations by introducing classical fluctuations of $f_{\text{Q1}} \to f_{\text{Q1}} + \delta f_{\text{Q1}}$, $f_{\text{Q2}} \to f_{\text{Q2}} + \delta f_{\text{Q2}}$ which are assumed to be static on the time-scale of 94 ns for a single Clifford gate. The fluctuations follow a Gaussian distribution with zero mean, standard deviation $\sigma_{\delta f_{Qj}}$ and are estimated from the corresponding dephasing times $T^{\star}_{2,\text{Qj}} = (2\sqrt{\pi}\sigma_{\delta f_{Qj}})^{-1}$ if not otherwise mentioned. To estimate the noisy density matrix we perform 250 noise implementations for each Clifford gate. In order to find the noisy superoperator we repeat the simulations for a full basis set of initial density matrices ρ_{init}. The noisy superoperator is then constructed via the linear system

$$\begin{pmatrix} \rho_{\text{final},1} \\ \rho_{\text{final},2} \\ \vdots \\ \rho_{\text{final},16} \end{pmatrix} = \mathcal{L} \begin{pmatrix} \rho_{\text{init},1} \\ \rho_{\text{init},2} \\ \vdots \\ \rho_{\text{init},16} \end{pmatrix}, \tag{44}$$

where $\mathcal{L}$ is the 16 × 16-dimensional superoperator. The noisy superoperator in Liouville form is then constructed by transforming into the basis $\rho_1 = |0\rangle\langle 0|, \rho_2 = |0\rangle\langle 1|, \rho_3 = |0\rangle\langle 2|, \cdots, \rho_{16} = |4\rangle\langle 4|$ following row ordering.

2. *Simulation of the Clifford experiments*

Since we now have the propagator or superoperator in Liouville form of each Clifford gate we can model a randomized benchmarking experiment. We model each randomized benchmarking protocol using the following procedure.

1. Create a sequence of N-Cliffords chosen randomly on both qubits and add the "recovery" Clifford to the sequence. For the standard two-qubit simultaneous RB protocol Cliffords the picked random Cliffords are uncorrelated between both qubits. For the two-copy RB protocol pick the same Clifford on both qubits. Repeat this K times.

2. Compute the final superoperator via multiplication of the corresponding two-qubit superoperator in Liouville form. This allows us to efficiently perform the initialization and measurement step at a later stage.

3. Chose an initial state and a measurement POVM to compute the expectation value for each sequence. Average over all K implementations.

4. Fit single exponential to the final data.

3. *Comparison between the results*

Supplementary Figures 6a and 6b show a comparison of the simulations of two-copy ES RB, two-copy ED RB, and SRB for different values of exchange interaction strength (6a), and dephasing times (T_{RB}) (6b). The simulations suggest that the Clifford fidelity of two-copy ES RB F_{2CRB} is larger than of SRB F_{SRB}. However, the difference of fidelities can be very small if the exchange interaction strength is smaller than 1 MHz. This can be explained that coherent errors, such as residual exchange interaction, only contribute to the average gate infidelity at second order [13]. However, reducing the amount of dephasing in the simulations shows that for J = 260 kHz the fidelity of the two protocols are still very close thus the dominant errors are most likely due classical cross-talk and not residual exchange interaction. We recommend when doing RB and in particular two-copy ED RB to characterize both qubits, as individual qubits may show non-monotonic fidelities as a function of noise. For example, in Supplementary Figure 6, we see an increase in fidelity for two-copy ED RB measuring qubit 2 for increasing interaction due to state distillation from the better to the worse qubit.

Supplementary figure 7 shows the simulated decay structure comparing four-copy RB (4CRB in Supplementary figure 7b) and four qubit simultaneous randomized benchmarking (4SRB in Supplementary figure 7b), for different values of qubit coherence (T_{RB}) for exchange interaction strengths of J_{12} = 260 kHz, J_{23} = 680 kHz, J_{34} = 340 kHz and J_{41} = 370 kHz, where J_{ij} is the exchange interaction strength between qubits Q_i and Q_j. These values are indicative of the exchange interaction strengths measured in the present system.

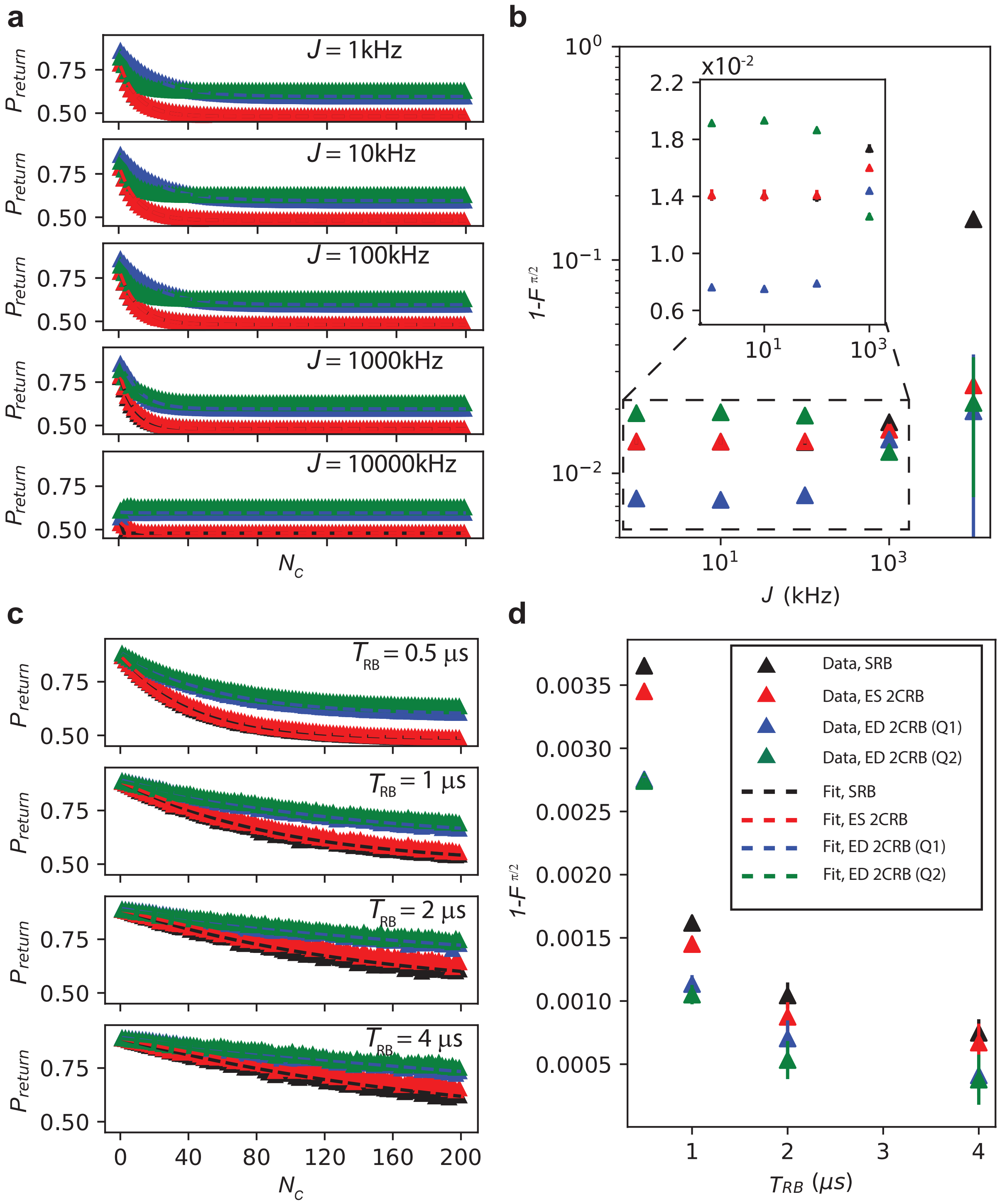

Supplementary Figure 6. **Full Hamiltonian simulations comparing two-copy ES, two-copy ED, and simultaneous randomized benchmarking**. (a) Simulated RB decay for two-copy ES (red), two-copy ED (blue), and simultaneous (black) randomized benchmarking protocols at different values of exchange interaction strength. (b) Extracted average generator fidelity. Divergence in performance occurs for values of exchange interaction strength above ≈ 0.5 MHz, where two-copy ES benchmarking returns higher average fidelities than SRB. (c) Comparing two-copy ES RB (red), two-copy ED RB (blue) and SRB (black) for different values of spin coherence time at $J = 260$ kHz. (d) Extracted fidelities for SRB, two-copy ES RB and two-copy ED RB, showing that all protocols return comparable fidelities at the exchange interaction strength present in our system. Error bars correspond to fit uncertainty.

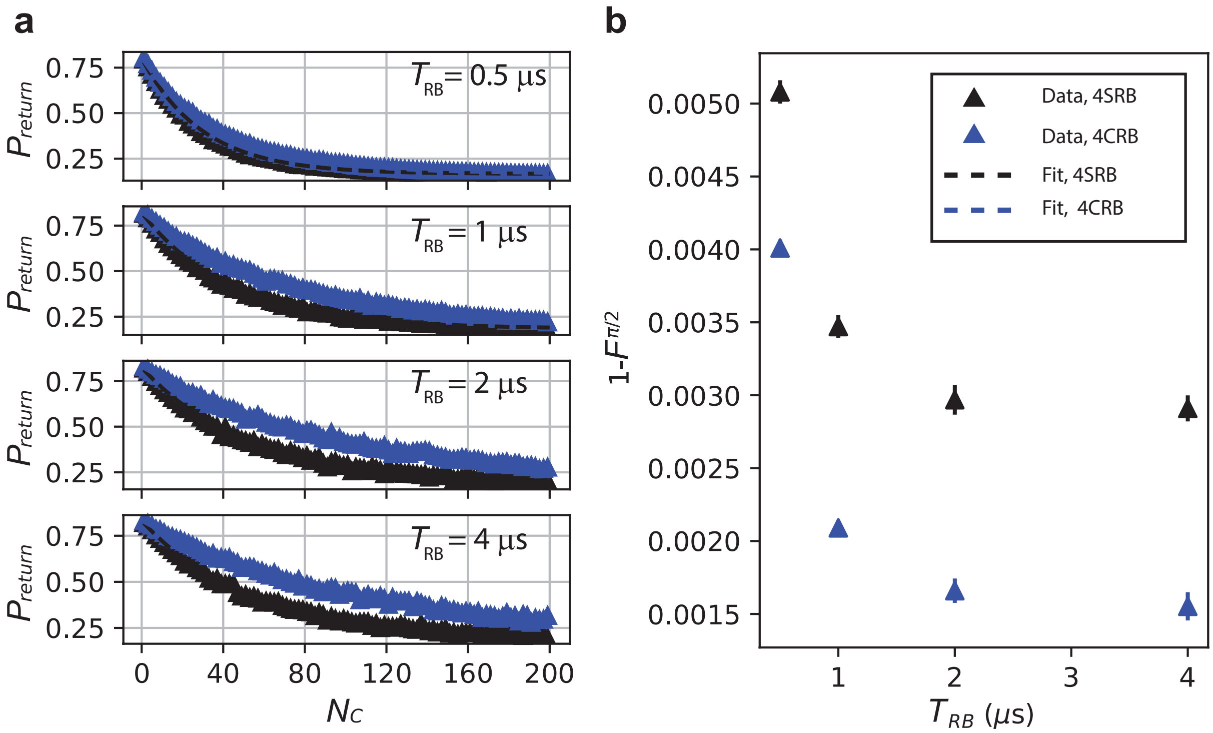

Supplementary Figure 7. **Full Hamiltonian simulations comparing four-copy and four-qubit simultaneous randomized benchmarking**. (a) Simulated RB decay for four-copy RB (red), four-qubit SRB (black) protocols at different values of qubit coherence time ($T^{2,RB}$). (b) Extracted average generator fidelity ($F^{\pi/2}$). Error bars correspond to fit uncertainty.

Supplementary Note 9: Fidelity Tables

Supplementary Table V. Single-qubit Clifford compilation for a direct comparison between two-copy and simultaneous benchmarking without idling times. We simulate both benchmarks by selecting a random sequence of Cliffords from the table below excluding C_1, and calculate the recovery Clifford that projects the system back into it's original state. We use here a gate set containing $\pm\pi/2$ rotations around the Bloch Sphere, so the gates $X_{\pm\pi/2}$ and $Y_{\pm\pi/2}$ are referring to explicit rotations of $\pm\pi/2$ around the x-axis and y-axis of the Bloch sphere of a single-qubit. $Z_{\pm\pi/2}$ are rotations around the z-axis and implemented "virtually" via software correction of the signal generator. In this respect a $Y_{\pi/2}$ rotation is implemented identical to a $X_{\pi/2}$ rotation with a $\pi/2$ software phase correction.

Clifford	Composition
C_1	$X_{\pi/2}X_{-\pi/2}$
C_2	$X_{\pi/2}X_{\pi/2}$
C_3	$Y_{-\pi/2}Y_{-\pi/2}$
C_4	$X_{\pi/2}X_{-\pi/2}Z_{\pi/2}Z_{\pi/2}$
C_5	$X_{\pi/2}Y_{-\pi/2}$
C_6	$X_{\pi/2}Y_{\pi/2}$
C_7	$X_{-\pi/2}Y_{-\pi/2}$
C_8	$X_{-\pi/2}Y_{\pi/2}$
C_9	$Y_{-\pi/2}X_{\pi/2}$
C_{10}	$Y_{-\pi/2}X_{-\pi/2}$
C_{11}	$Y_{\pi/2}X_{\pi/2}$
C_{12}	$Y_{\pi/2}X_{-\pi/2}$
C_{13}	$Y_{-\pi/2}X_{\pi/2}Z_{-\pi/2}$
C_{14}	$Y_{-\pi/2}X_{-\pi/2}Z_{\pi/2}$
C_{15}	$X_{\pi/2}Y_{-\pi/2}Z_{\pi/2}$
C_{16}	$X_{\pi/2}Y_{\pi/2}Z_{-\pi/2}$
C_{17}	$X_{-\pi/2}X_{\pi/2}Z_{\pi/2}$
C_{18}	$X_{-\pi/2}X_{\pi/2}Z_{-\pi/2}$
C_{19}	$X_{\pi/2}Y_{-\pi/2}Z_{-\pi/2}$
C_{20}	$X_{\pi/2}Y_{\pi/2}Z_{\pi/2}$
C_{21}	$Y_{-\pi/2}X_{\pi/2}Z_{\pi/2}$
C_{22}	$Y_{-\pi/2}X_{-\pi/2}Z_{-\pi/2}$
C_{23}	$X_{\pi/2}X_{\pi/2}Z_{\pi/2}$
C_{24}	$X_{-\pi/2}X_{-\pi/2}Z_{-\pi/2}$

Q_i	$F_{Qi}^{\pi/2}$	F_{Qi}^{C}	t_π	B_{ext}
Q1	99.980(4)	99.94(2)	97 ns	0.65 T
Q2	99.936(9)	99.79(3)	71 ns	0.65 T
Q3	99.992(1)	99.974(3)	58 ns	0.65 T
Q4	99.7(2)	99.0(6)	41 ns	0.65 T
Q1	99.989(1)	99.965(3)	50 ns	1 T
Q2	99.90(2)	99.68(6)	48 ns	1 T
Q3	99.958(2)	99.865(6)	50 ns	1 T
Q4	99.79(2)	99.32(6)	50 ns	1 T

Supplementary Table VI. Summary of best single qubit fidelities and tuning parameters at B_{ext} = 0.65 T and B_{ext} = 1 T. Quoted errors correspond to the fit uncertainty.

Q_iQ_j	$F_{QiQj}^{\pi/2}$	F_{QiQj}^{C}	t_π	B_{ext}
Q1Q2	99.72(1)	99.10(3)	69 ns	0.65 T
Q3Q4	99.905(8)	99.69(3)	61 ns	0.65 T
Q1Q2	99.81(3)	99.4(1)	35 ns	1 T
Q3Q4	99.52(4)	98.5(1)	50 ns	1 T

Supplementary Table VII. Summary of best ES two-copy RB fidelities and tuning parameters at B_{ext} = 0.65 T and B_{ext} = 1 T. Quoted errors correspond to the fit uncertainty.

Q_iQ_j—Q_kQ_l	$F^{\pi/2}_{QiQj\|QkQl}$	$F^{C}_{QiQj\|QkQl}$	t_π	B_{ext}
Q1Q2—Q3Q4	98.9(2)	96.5(6)	150 ns	0.65 T
Q3Q4—Q1Q2	99.0(1)	96.8(3)	150 ns	0.65 T
Q1Q2—Q3Q4	99.3(2)	97.7(6)	60 ns	1 T
Q3Q4—Q1Q2	99.34(4)	97.9(1)	55 ns	1 T

Supplementary Table VIII. Summary of best ES four-copy RB fidelities and tuning parameters at B_{ext} = 0.65 T and B_{ext} = 1 T. Quoted errors correspond to the fit uncertainty.

[1] Helsen, J.; Roth, I.; Onorati, E.; Werner A.H.; Eisert, J. *PRX Quantum* **2022,** *3,* 020357
[2] Wallman, J. J.; Flammia, S. T.; *New Journal of Physics* **2014,** *16,* 103032.
[3] Helsen, J.; Wallman, J. J.; Wehner, S.; *Journal of Mathematical Physics* **2018,** *59*, 072201.
[4] Zhu, H.; Kueng, R.; Grassl, M.; Gross, D; **2016,** *arXiv:1609.08172 [quant-ph].*
[5] Li, W.; Liao, W.; Fannjiang, A.; *IEEE Transactions on Information Theory,* **2020,** *66* 4593-4608.
[6] Helsen, J.; Battistel, F. Terhal, B. M. *npj Quantum Inf,***2019,** *5* 74.
[7] O'Brien, T. E.; Tarasinski, B.; Terhal, B M. *New Journal of Physics,* **2019,** *21. 023022*.
[8] Wallman, J. J.; **2015,** *arXiv:1511.00727 [quant-ph].*
[9] Xue, X.; Watson, T. F.; Helsen, J.; Ward, D. R.; Savage, D. E.; Lagally, M. G.; Coppersmith, S. N.; Eriksson, M. A.; Wehner, S.; Vandersypen, L. M. *Physical Review X* **2019,** *9*, 021011.
[10] Hendrickx, N. W.; Lawrie, W. I.; Russ, M.; van Riggelen, F.; de Snoo, S. L.; Schouten, R. N.; Sammak, A.; Scappucci, G.; Veldhorst, M. *Nature* **2021,** *591*, 580—585.
[11] Hetényi, B.; Kloeffel, C.; Loss, D. *Physical Review Research,* **2020,** *2,* 033036.
[12] Meunier, T.; Calado, V. E.; Vandersypen, L. M. K. *Phys. Rev. B,* **2011,** *83,* 121403.
[13] Blume-Kohout, R.; da Silva, M. P.; Nielsen, E.; Proctor, T.; Rudinger, K. Sarovar, M.; Young, K. *PRX Quantum,* **2022,** *3,* 020335.
